# DBpedia Archivo: A Web-Scale Interface for Ontology Archiving Under Consumer-Oriented Aspects

**DOI:** 10.1007/978-3-030-59833-4_2

**Published:** 2020-10-27

**Authors:** Johannes Frey, Denis Streitmatter, Fabian Götz, Sebastian Hellmann, Natanael Arndt

**Affiliations:** 8 Linköping University, Linköping, Sweden; 9 University of Amsterdam, Amsterdam, Noord-Holland The Netherlands; 10 Department of Computer Science, Vrije Universiteit Amsterdam, Amsterdam, Noord-Holland The Netherlands; 11 St. Pölten University of Applied Sciences, St. Pölten, Austria; 12 FIZ Karlsruhe – Leibniz Institute for, Karlsruhe, Germany; 13 Karlsruhe Institute of Technology, Karlsruhe, Germany; 14 UAS St. Pölten, St. Pölten, Niederösterreich Austria; 15 Vienna University of Economics and Business, Vienna, Wien Austria; 16 VU Amsterdam, Amsterdam, The Netherlands; 17 ADAPT Centre, Trinity College Dublin, Dublin, Ireland; InfAI and Leipzig University, AKSW, Leipzig, Germany

**Keywords:** Ontology archive, Ontology repository, Ontology crawling

## Abstract

While thousands of ontologies exist on the web, a unified system for handling online ontologies – in particular with respect to discovery, versioning, access, quality-control, mappings – has not yet surfaced and users of ontologies struggle with many challenges. In this paper, we present an online ontology interface and augmented archive called DBpedia Archivo, that discovers, crawls, versions and archives ontologies on the DBpedia Databus. Based on this versioned crawl, different features, quality measures and, if possible, fixes are deployed to handle and stabilize the changes in the found ontologies at web-scale. A comparison to existing approaches and ontology repositories is given .

## Introduction

Phrases such as “A little semantics goes a long way”^1^ or “Let a thousand ontologies blossom” [7] have shaped the landscape of ontologies on the Semantic Web. Ontologies are the common language spoken on the Semantic Web, they represent schema knowledge and provide a common point of integration and reference while the value of an ontology grows with its use. As the conceptual framework to globally interlink distributed knowledge, ontologies provide the backbone of the Semantic Web.

While thousands of ontologies exist on the web, a unified system for handling online ontologies has not yet surfaced and both publishers and users of ontologies struggle with many uncertainties and challenges. The main discussion and effort so far in the Semantic Web community is unbalanced and focused on authoring and publication of ontologies and linked data in general with serious consequences. The community produced several guidelines, rules, methodologies and tooling for publishers neglecting users and clients. However, the variety increases uncertainty by offering too many choices, increases effort and complexity through the need to understand and implement several guidelines and provides no or unclear incentives or rewards to the publisher to comply with them.

As a consequence, the consumer is left to deal with the resulting heterogeneity, quality issues and failures. The majority of problems and challenges fall into the categories access and quality. We have identified several *Usage Challenges* which we enumerate in parentheses for reference in the remainder of the paper. Major *physical* access problems are caused by link rot (UC1) and incorrect Linked Data deployments (UC2), but most crucially there is no established, stable citation or dependency system for ontologies like Maven or DOI (UC3) - ontologies or parts of it can change or disappear anytime. Additionally, heterogeneity increases the complexity to access ontologies. There can be no, unclear or inconsistent versioning (UC4a), the versioning nomenclature can substantially vary (UC4b) and guarantees w.r.t. backward-compatibility usually remain unclear (UC4c). Various formats to serialize OWL ontologies exist (e.g. OWL-XML, RDF-XML, Manchester Syntax, Turtle; UC5). In case that an application/consumer succeeded to retrieve an ontology (version) several quality problems can prevent proper processing/usage. Parsing of the RDF snapshot can fail (UC6), problems w.r.t. licensing can prevent the usage at all (UC7) due to missing, unclear, heterogeneous (several properties and license IDs) or too restrictive or improper licensing. Finally, the fitness for use can be limited due to low quality metadata (e.g. missing labels, title; UC8) or logical inconsistencies (UC9).

In this paper, we present a web-scale ontology interface called DBpedia Archivo (acronym for ontology archive), that discovers, crawls and versions ontologies and archives as well as augments them on the DBpedia Databus [5]. The primary purpose of this interface is to help users/consumers to discover, access and validate/assess the quality/usability of ontologies in a unified way, while reducing the challenges and effort to spot and deal with the mentioned issues, such that they can focus on building stable and reliable applications. Nevertheless, we also aim to support both the consumer and the publisher by augmenting the ontology (e.g. reporting quality metrics, generating documentation). We envision in the mid/long-term, that with the help of Archivo we foster the adherence to standards (publicly showing issues, basic quality control for access to Archivo) and strengthening incentives for publishers (bad metadata e.g. no dct:title, dct:description results in worse findability and presentation in Archivo), such that the overall quality of the ontologies in the Web of Data emerges, which in return would benefit users and applications.

**Fig. 1.**
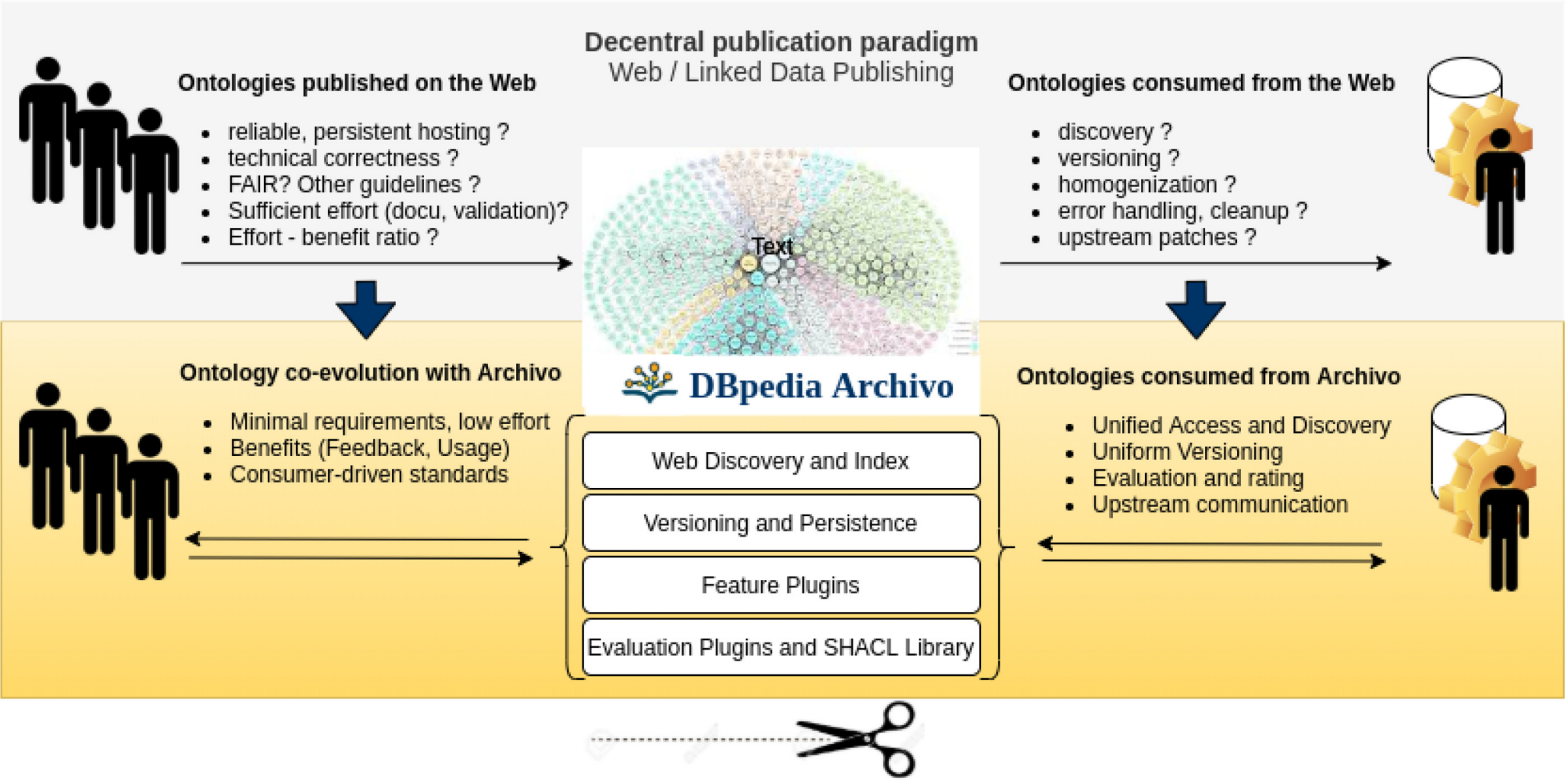
Interface and platform model of Archivo

We argue, that a crucial factor for the success of the web were working web browsers and search engines that increased user numbers and views and created incentives to publish correct and high quality websites. Following this line, as a novel paradigm, DBpedia Archivo (see Fig. 1) proposes a consumer/application-oriented approach to the Semantic Web.

At a glance, with DBpedia Archivo we make the following **contributions**:

1. Discovery (including user suggestions), crawling, versioning, archiving and evaluation of ontologies with a high degree of **homogenization and automation**,
2. unified, stable, referenceable **identifiers for each ontology version**, so that ontology consumption becomes stable and applications, experiments and research with a specific version of an ontology, can be reproduced at any time,
3. unified time-based and Semantic Versioning enabling **auto update applications** with custom trade-off between latest changes and stability (user controlled up-to-dateness),
4. the augmented archive includes add-ins and extensions which enhance the use of an ontology, among others, generated documentation, **quality reporting** with a consumer-oriented star rating and results of validation and test steps.

In the following section we provide an overview on related work. In the subsequent section we briefly introduce the conceptual ideas of Archivo and its platform model. Sect. 4 describes the implementation. In Sect. 5 we introduce an automatically verifyable consumer rating. An evaluation of an initial crawl of ontologies based on our rating as well as a comparison of Archivo to existing ontology repositories is given in Sect. 6.

## Related Work

Related work can be separated into three areas: archiving and versioning tools for ontologies, ontology repositories (which are compared in depth to Archivo in Sect. 6) and ontology validation and testing tools.

### Archiving and Versioning

The **Memento** protocol [19] allows to discover and browse old versions (Mementos) of web resources. The Internet Archive provides a prominent service, WaybackMachine,^3^ a generic archive for web resources (including a subset of ontologies from the web) accessible using Memento. Moreover, Memento is used and adapted by the **TailR** system [11], a self-deploy/service archiving system for Linked Data resources and the **Triple Pattern Fragment Server** which can be used to serve and query archived Linked Data [21] with lower infrastructural efforts. Unfortunately, Memento is currently not (widely) adopted for ontology publication and to the best of our knowledge, there is no support for Memento in ontology tools, yet. Archivo offers with SPARQL and Linked Data well-known, standardized and with the help of DataID metadata a unified way to discover, access but also query (relevant) versions of an ontology but additionally serves as a central point to discover (archived) ontologies. Realization of Memento on top of Archivo is possible and subject to future work.

**SemVersion** [22] proposes a methodology and Java API for RDF (and ontology) versioning inspired by CVS. It offers a structural and a form of semantic diff between two versions, achieved by performing structural diffs on semantic closures (RDF(S) entailment). The semantic diff of Archivo based on (OWL) axiom diffs goes a step further. **Quit** [2] implements an RDF versioning and collaboration system on top of Git. It provides unified access via SPARQL 1.1 on each version of an ontology and the versioning history. Both systems focus on ontology development rather than the consumer perspective.

**D2V** is a tool to manage and visualize user-defined changes in RDF data. In [17] it is demonstrated for ontology evolution measuring specific types of changes (e.g. added properties / labels or deprecated classes). While D2V allows very flexible, use-case/dataset specific-analysis of changes, Archivo’s additional Semantic Versioning aims at making the trade-off between unified and flexible/fine-grained change reports with 3 types of changes (major, minor, patch).

**Vocol** [6] is an integrated environment based on Git and several services to enable collaborative vocabulary development. The workflow consists of 3 activities: modeling, population and testing (syntactic and semantic validation, competency questions), deployment of ontologies (machine- and human-readable). While some of the features (semantic diff and validation, documentation generation, custom tests for ontologies) are similar to Archivo, Vocol was designed for publishers, consumers depend on them to take advantage of the system.

### Ontology Repositories and Platforms

There have been ample efforts to provide a platform, repository, library or other web services to deal with storage, search, retrieval of ontologies, some of which do not exist or work properly anymore. For reasons of brevity, we only mention approaches which are, to the best of our knowledge, still active and functional. We refer the reader to [4] for a time travel to a decade ago.

In our scope we identify 4 major characteristics of such systems. An archive persists ontologies (and its versions). A catalog associates a list of ontologies with thorough metadata. As index we denote a system that allows to search components (e.g. classes) of ontologies. A development platform is a workspace with integrated tools to create and handle ontologies.

**OntoMaven** [13] is a distributed ontology archiving approach based on the maven philosophy. Ontologies and its dependencies are organized in mvn artifacts. As a consequence transitive imports can be resolved and downloaded locally. A set of mvn plugins supports several aspects of ontology development lifecycles, e.g. import, creation of documentation and reports, consistency tests and versioning. Although we were not able to find any announced public repository, the ontology organization structure is very similar to the one of DBpedia Databus [5] Archivo is based on.

**OBOFoundry** [18] is an ontology developer initiative in the biological and biomedical domain which manually curates a catalog of approved ontologies. The registering of new ontologies follows a set of design principles (e.g. naming convention, versioning strategy) which are verified semi-automatically. The foundry operates its own PURL service to offer stable identifiers.

**BioPortal** [23] is another prominent catalog in the biomedical domain. It offers storage for ontology submissions and archiving to registered users and performs indexing on the latest submission. Moreover, it offers developer platform features such as user access rights and mappings between ontologies.

Linked Open Vocabularies [20] (**LOV**) is a semi-automatically curated catalog of vocabularies. It offers a search index on the terms defined in the vocabularies, a SPARQL Query endpoint and provides persistent access to the history of vocabularies. New vocabularies are discovered by analyzing (re)use of terms from archived ontologies or can be suggested by users.

**Ontobee** [12] creates an index for OBOFoundry and a portion of other ontologies. It serves the ontologies as linked data and provides search and browsing interfaces. Another index in the biomedical domain is the **Ontology Lookup Service** [10].

**OntoHub** [3] is an open ontology repository engine with versioning based on Git following Open Ontology Repository Initiative (OOR) requirements. It offers homogeneous formal representation of ontology axioms using DOL, testing with HETS and competency questions. An instance of it operates ontohub.org which is free to users and contains a plethora of ontologies, including imports from other repositories.

### Ontology Evaluation and Validation

The list of literature with rules and guidelines to follow is extensive. We would like to list [9, 15, 24], the LD principles,^4^ LOD Cloud,^5^ LOV^6^ and refer to their references for brevity. We picked the prominent **Ontology Pitfall Scanner! (OoPS!)** [16], also used by Archivo, as a representative for the many existing validation & evaluation approaches as it provides an excellent overview of other literature. **OnToology** [1] is a service (based on OOPS and other tools) to create pull request for ontologies hosted on GitHub to deliver test reports and documentation. It is similar to the ontology augmentation concept of Archivo, however needs to be configured and managed by the repository owner/publisher.

**ROBOT** [8] deserves a special mention as a highly automatized and configurable evaluator. The idea here is that sub-communities for certain domains (e.g. biological and -medical) configure and deploy the tool for their community. While similar (configure local needs, deploy local), Archivo follows a more generic approach (configure local needs, deploy global).

## Archivo Platform Model

### Versioning and Persistence on the Databus

DBpedia Archivo is built on top of the DBpedia Databus [5], which is inspired by Maven Central Repository. It uses the maven concepts publisher/group/artifact/version and ports them to a Linked Data platform, in order to manage data pipelines and enable automatic publishing and consumption of data.

Archivo is a dedicated publishing agent on the Databus.^7^ Similar to [13] artifact IDs (represented as IRIs) are used as stable identifiers to reference an ontology with no regard to its evolution (UC1 and UC3). A version string appended to the artifact IRI forms a stable ID to resolve a particular version. An extension of the DataID metadata vocabulary for artifact, version, and files allows for flexible and fine-grained access using SPARQL. The concepts of time-based (UC4a/b) and semantic versioning (UC4c) support increased stability of applications while allowing automatic updates to some (user-configurable) degree.

Databus file identifiers form a stable abstraction layer independent of hosting and similar to PURL by using dcat:downloadURL links in the metadata. Crawled ontologies and metadata are persisted on the DBpedia download server^8^. Creating a mirrored archive of ontology versions such as Archivo is, of course, not infallible. We consider it, however, a sufficiently reliable fall-back to improve persistence of ontologies on the Semantic Web.

### Evaluation Plugins and SHACL Library

DBpedia Archivo largely builds on the W3C SHACL^9^ standard. While minimal basic validation as described in Sect. 5 is fixed (part SHACL, part code), the remaining validation is done via a SHACL library that is partitioned into SHACL test suites for specific purposes: 1) they can encode general validation rules (e.g. from OOPS and tackle UC7), 2) they can capture specific requirements needed by Archivo features such as the automatic HTML documentation generation of LODE (UC8) (cf. next section), 3) they can be sub-community or use case-specific down to individual user projects. While at the time of writing few SHACL test suites exist, we allow online contribution and extension (Validation as a Platform) for Archivo to run in the hope to give consumers a central place to encode their requirements and also discuss and agree on more universal ones.

### Feature Plugins

Feature plugins in DBpedia Archivo augment a certain aspect of the ontology, e.g. generate documentation, visualization or automatic mappings. While a complete overview is out of scope of this paper, we integrated the Live OWL Documentation Environment (LODE) [14] into Archivo, which generates a uniform HTML documentation for each version of all archived ontologies. Adding more features is straightforward. Pre-generated results make them universally available for all ontologies and absolve publishers and consumers to find, learn and deploy such ontology tools.

## Archivo Implementation

The guiding principle for Archivo’s implementation follows Jon Postel’s law: “Be conservative in what you do, be liberal in what you accept from others”. Being “liberal” in the context of Archivo has clear limits. While we accept ontologies in different formats, work around small mistakes (e.g. also recognizing incorrect dc:license triples instead of dct:license) (UC8) and even use recovering parsers that can skip syntax errors (UC6), we decided to be strict in all aspects that directly contradict the automatic processing of ontologies and therefore either heavily impact their usefulness or require meticulous archaeological excavation work to use and archive them. Since we invested the time to implement the most common retrieval and processing methods, our guideline is **“If DBpedia Archivo can not process it in an automatic and deterministic manner, it is likely infeasible to be processed”** based on the assumption that the Semantic Web was created for machines. One prominent example here is the missing license declaration in the FOAF RDF/XML document,^10^. While the HTML documentation includes the license using RDFa,^11^ it only yielded 348 triples, compared to 631 in RDF/XML. While staying “liberal”, there is no optimal automatic choice on what to accept: half the ontology with license, full ontology without license. Our strategy is that we are liberal at the launch of Archivo to allow old/unmaintained (but potentially already widely used) ontology versions to be archived but we will become more restrictive (no archiving of new ontology (versions) that do not fulfill baseline criteria) after an establishing phase. The strictness in such cases stems from the rationale that these non-automatic and non-deterministic ontologies will eventually **cause an immeasurable and unacceptable amount of effort in the downstream network of consumers**.

### Ontology Discovery and Indexing

**Fig. 2.**
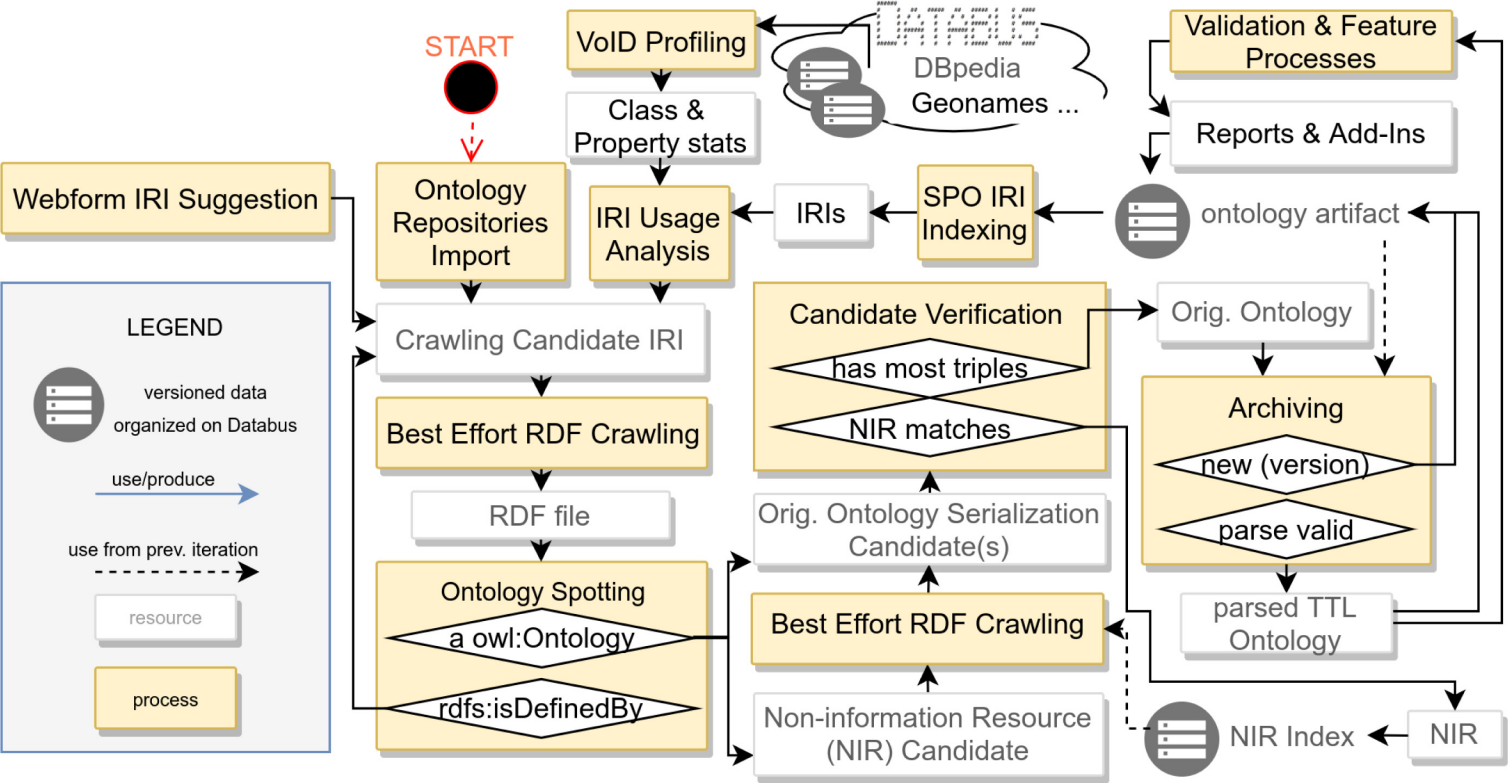
Overview of iterative ontology discovery and archiving

The goal of the discovery and indexing phase is to create a distinct set (index) of non-information URIs/resource (NIR) of ontologies for each iteration as input for further crawling and processing. We devised four generic approaches to feed Archivo with ontology candidates (crawling candidate IRIs) and implemented them as a proof-of-concept.

**Ontology Repositories:** One straightforward way of retrieving ontology URIs is by querying already existing ontology repositories. The repository with the broadest collection of very popular ontologies of the Linked Open Data Cloud is Linked Open Vocabularies (LOV) [20], which we used in this paper. LOV provides a simple API which contains (among other metadata) candidates for non-information URIs.

**Vocabulary Usage Analysis via VoID:** Another approach to discover ontology candidates is by analyzing vocabulary usage in the data. Our goal here is in particular to cover all vocabularies used by datasets uploaded onto the Databus, which already contains several datasets besides DBpedia, such as Geonames, Caligraph, MusicBrainz and the German National Library, just to name a few. As the Databus provides a controlled and harmonized environment, we generate a virtual class-based and property-based partition^12^ for all RDF files on the bus, thus retrieving a list of all classes and properties.

**Discovery via Links to External Ontologies:** As Archivo already creates a controlled and harmonized ontology archive, we can exploit the refined collection of ontologies from the previous iteration to discover further ontology candidates. For this purpose, we extract a list of all subject, predicate and object IRIs from the ontologies itself to create more leads to properties/classes/ontology files.

**Manual Suggestion:** Automatic discovery is able to capture and persist most of the currently available ontologies in a forward-progressing manner. In addition manual/external suggestions of ontology candidate IRIs are accepted via web form^13^ to increase Archivo’s coverage and to offer an on-demand archiving function (UC3). Moreover, we consider this feature helpful for ontology engineers to test and receive feedback already during the development phase.

Subsequent to the aforementioned discovery steps we crawl/check every candidate IRI. The best effort crawling tries to download multiple RDF files via different HTTP-accept headers (in case a *robots.txt* is not disallowing access for the Archivo crawler) (UC2 and UC5). At the time of writing two additional rules are in place for considering an ontology/vocabulary as valid candidate for inclusion into Archivo: 1) the NIR needs to resolve to an RDF document rapper can read, 2) we require the existence of an entity identified by the NIR which is typed as owl:Ontology or skos:ConceptScheme (which should carry additional metadata and makes the ontology spottable in reliable way) in the triples output of the failure-tolerant parser. If multiple valid serialization candidates exist, we give preference to the serialization having the highest triple count (this will archive the correct FOAF version without license). Finally, the NIR is appended to the index and the chosen serialization is passed over for a release on the Databus. If the spotted NIR doesn’t match with the candidate IRI it started with, the retrieved NIR becomes a new NIR and the process starts again (see Fig. 2). The crawling candidate IRIs representing properties and classes with a slash URI scheme require a special treatment in case the resolution does not return the ontology itself. We use skos:inScheme and rdfs:isDefinedBy as pointers to a new candidate IRI.

### Analysis, Plugins and Release

**Analysis and Integration of Feature Plugins:** In every new snapshot, we augment the original ontology file with a parsed ntriples, turtle and owl version to simplify the access (UC5 and UC6). Additionally, to the plugins and validation methods described in Sect. 3, the reasoner Pellet^14^ is used for checking the consistency (UC9) of the ontology and determining the OWL profile. Furthermore an OOPS report (UC8) is generated to detect common pitfalls of the ontology. All reports are stored alongside the original snapshot with appropriate DataID metadata to augment the snapshot.

**Release on the Databus:** To deploy an ontology on the Databus we use its non-information URI as the basis for the Databus identification. The host information of the ontology’s URI serves as the groupId and the path serves as the name for the artifactId. Archivo’s lookup component^15^ with Linked Data interface allows to resolve the mapping from a non-information URI to the stable and persistent Databus identifier.

### Versioning and Persistence

**Time-Based Snapshots:** For all verified non-information URIs in the index, Archivo looks for new versions a few times each day. To reduce the amount of transferred data, Archivo uses the HTTP-headers E-Tag, Last-Modified and content-length to detect via a HEAD-request if the respective ontology resource could have changed. If any of the headers changed (or if none of the headers is available), the vocabulary is downloaded and checked locally for changes.

The local diff is performed by converting the downloaded source with rapper^16^ to canonical N-Triples, sorting them and comparing them with comm^17^ to determine if any triple was added or deleted. This process requires the new version to be parseable without errors. In case a change could be verified the new snapshot is released with using the fetch timestamp as version label.

**Semantic Versioning:** If a change in the set of triples was detected, a set of (description) logic axioms is generated for both the old and new version of the ontology and those axioms are compared to each other. In case of no changes in the axioms, no structural ontology change was done (e.g. added only labels, or ontology metadata) the change is classified as patch. If only new axioms were added, we consider this as a new minor version. If new classes/properties are added, this usually leads to no backward-compatibility problems for existing applications, but there are cases (e.g. adding a deprecated or disjoint relation to a class) which might have consequences in combination with A-boxes. Any deletion of already existing axioms (thus including renaming) is considered as major change potentially seriously affecting backward-compatibility. This semantic versioning “overlay” allows a more fine-grained update decision than the binary “take it or leave it” (UC4a-c). Users can refine the trade-off with custom solutions based on the semantic versioning and axiom diffs. We plan that more sophisticated versioning overlays can augment the Archivo snapshots with open contributions via Databus mods (see Sect. 7).

## A Consumer-Oriented Ontology Star Rating

Following the argumentation of Sect. 4 our proposed rating system is “liberal” to a certain degree of heterogeneity, but strict in the sense that it awards low ratings to ontologies that defy automatic or deterministic processing. The proposed star rating differs from written rules and guidelines in human language in these aspects: 1) stars are formalized and algorithmically verifiable and can be tested, 2) they are executed over the known, ontological part of the Semantic Web captured in Archivo and are meant to be delivered to consumers to quickly assess the technical usability and soundness 3) they are centrally available, frequently executed, debatable and extendable. They allow capturing and crowd-sourcing of consumer needs. We included short references to other approaches from [16]^18^ (integrated, see below), [8]^19^ and [9] (VocUse, partly applicable). From DBpedia Archivo perspective, some requirements become redundant such as the HTML documentation, which can be generated, if the appropriate SHACL test is successful. Others become more strict (machine readability).

### Two Star Baseline

We consider the two star baseline as a minimal requirement for considering the ontology as a legit participant in the Semantic Web. An ontology which does not fulfill the baseline can’t earn any further stars.

$$\star $$
**Retrieval and Parsing**: All of the following criteria have to be fulfilled: (1) The non-information URI resolves to a machine readable format or a machine readable version is deterministically discoverable by other common means, (2) download was successful, (3) uses a common format implemented by Archivo, (4) at least one format was found that parses with no or few (negligible) syntactical warnings (UC6). [OBO fp2, OOPS! P37, VocUse 2]
$$\star $$
**License I**^20^: A proper ontology declaration was found using a owl:Ontology and some form of license could be detected. A high degree of heterogeneity is permissible for this star regarding the used property/subproperty as well as object: license URI (resolvable linked data or web link), xsd:string or xsd:anyURI (UC7). [OBO fp1, OOPS! P38 P41, VocUse 4]

### Quality Stars

On top of the two star baseline, Archivo implements additional criteria. The main rationale behind these stars is to ease effort for client implementations by homogenizing the retrieved data and the technical expectations a client can have towards mirrored ontologies by Archivo.

$$\star $$
**License II**: We require a homogenized license declaration using dct:license as object property with a URI (not string or anyURI). If a resolvable Linked Data URI is used, we expect the URI to match the URI used in the machine readable license (UC7). We discovered many irregularities such as trailing ‘/’ which violate RDF requirements that URIs need to be exactly the same in RDF as opposed to Linked Data resolution. In the future, we plan to tighten up this criterion and expect machine readable license, which we will collect on the DBpedia Databus in a similar manner as Archivo. [OBO fp1, OOPS! P41, VocUse 4]
$$\star $$
**Logical Fitness**: Although logical requirements such as consistency are theoretically well-defined, from a consumer perspective this star is highly implementation-specific. We measure the compatibility with currently available reasoners such as Pellet/Stardog (more to follow) and run available tasks such as consistency checks (UC9), classification, etc. since owl:disjointWith axioms are nice, unless they render the ontology unusable for reasoning.

**Fig. 3.**
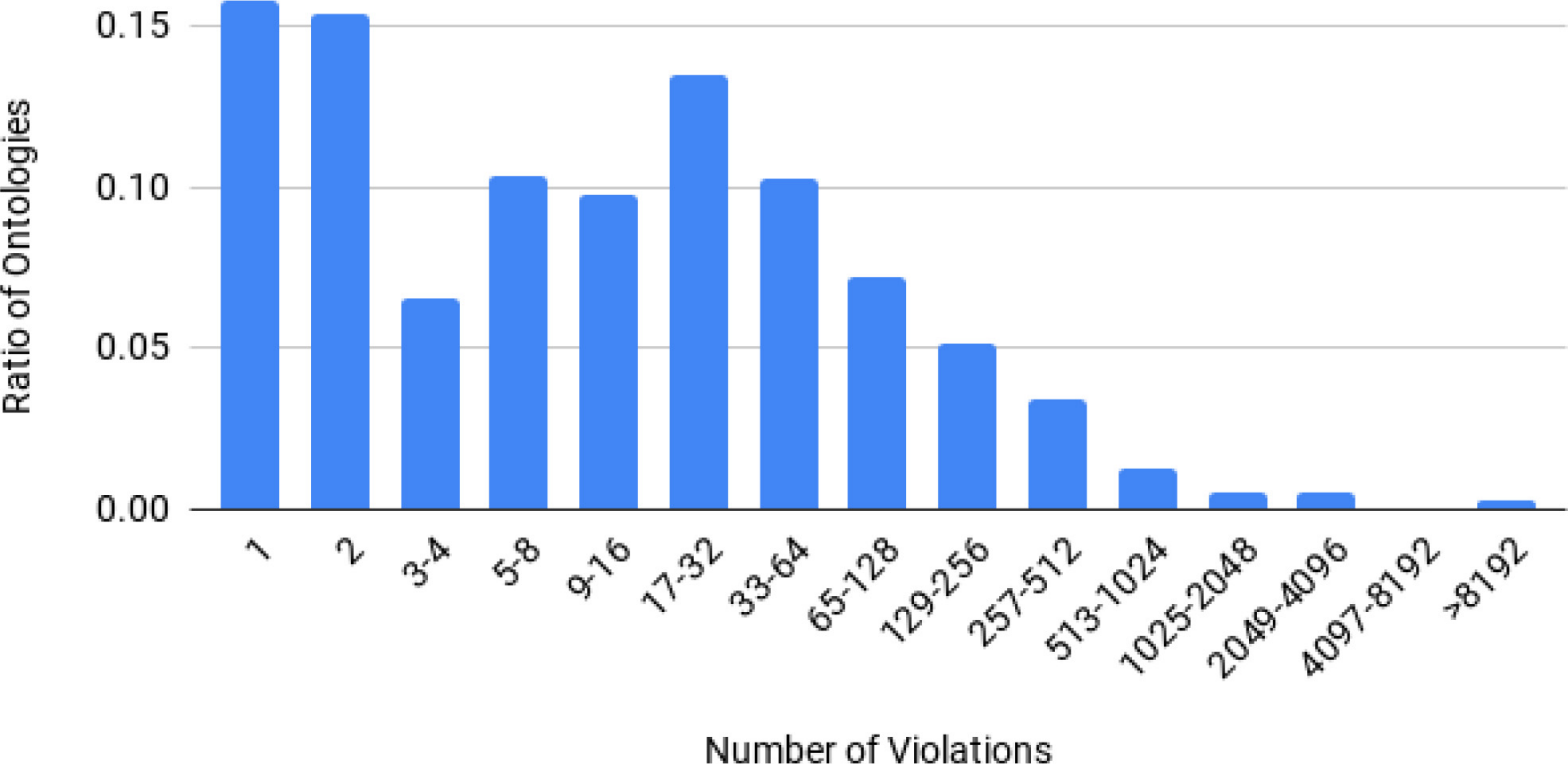
Distribution of violations per ontology using SHACL-based LODE tests

**Table 1.** Results for Archivo (July 2020) testing and rating

#Ont.	Stars^1^	License-I^2^	License-II^2^	Consistency^2^	LODE^3^	Expressivity^4^
735	11/453/10/134/127	275/460/0	137/598/0	687/23/25	1/30/702/2	103/91/9/29/15/488

^1^Format: 0/1/2/3/4 Stars ^2^Format: True/False/Error ^3^Format: OK/Warnings/Violations/Error

^4^Format: OWL2 FULL/DL/QL/EL/RL/Tool Error

### Further Stars and Ratings

We practiced a large amount of self-discipline not to encode more stars with our ideas and opinions as they didn’t pass our own relevancy criteria (Who needs this?). Further stars and ratings could provide direct incentives for ontology publishers such as the ability to generate HTML documentation with LODE (tested with SHACL) or represent user needs, or could be of analytical nature, such as adoption and re-usage (inbound links from other ontologies and data, [9] VocUse 3 and 5).

## Evaluation

### Archivo and Rating Statistics

DBpedia Archivo consists of 735 ontologies in July 2020. The biggest fraction of it (401) was discovered via the LOV-API, 268 were discovered from prefix.cc and the rest was retrieved from the subjects, predicates and objects of the ontologies in Archivo itself (60) and user suggestions (6). Unfortunately the Usage Analysis via VOID didn’t yield any new ontologies, but this feature was added at last, so the index already contained the used ontologies of datasets from the Databus. Figure 3 shows the ratio of ontologies that share a class of violations numbers. The diagram shows that, even though a small amount of ontologies are quite badly curated, the biggest share of ontologies has quite low error numbers, allowing a smooth generation of LODE documentation. Table 1 shows that more than 60% of the ontologies have less than two stars. Almost every one star rating is caused by a missing license. Since an open license is a fundamental requirement of open data, it is a bad sign for the usability of the available ontologies on the web. With more than 90% of logical consistency the ontologies are sitting pretty, but as mentioned this value can be highly implementation specific.

### System Comparison

We identified 7 other (ontology repository) systems which are either very similar on a conceptual or technical level (e.g. LOV, OntoMaven) or are active systems which serve a notable set of ontologies to users. While the type and primary usage of the systems vary, we assessed them under a common set of features along the 4 dimensions coverage, recency, access and quality (see Table 2). While access and quality dimensions stem from the problem analysis, a sound strategy for both a high coverage and recency w.r.t. archived ontologies seem natural requirements from the perspective of users and tools demanding for one unified solution to efficiently tackle the problems. We argue that such a system needs to offer and be built on a high level of automation and homogenization (unified and standardized/well known practices) to successfully tackle web-scale dimensions and (if done correctly) optimize client side processes (decreased consumer side effort and increased usage benefits). We selected features reflecting this.

**Table 2.** System (feature) comparison along the dimensions coverage, r(ecentness), access and (q)uality.

Dimension		Coverage			r	Access						q
System name	TY	DO	IM	DI	UP	UV	SV	ID	PE	OF	MA	TE
Archivo	A	I										
Bioportal	all	S	^1^	-	^2^		-	^1^		-		-
LOV	C,A,I	I					-					-
OBO foundry	C	S	-/-	-	-		-		-		-/-	
Ontobee	I	S	-/-	-		-	-	-	-			-
Ontohub.org	D	I	^1^	-	^3^	-	-				^4^/-	
OntoMaven repo	A	-	^1^	-	-	-	-				^5^/-	
Ont. Lookup Svc	I	S	-/-	-		-	-	-	-	^6^		-

Dash represents *no*, white/black filled circle represent *partial*/*full* support; **TY**: system type - (A)rchive, (C)atalog, (I)ndex, (D)evelopment Platform; **DO**: ont. domain focus - (S)pecialized vs. (I)ndependent; **IM**: ont. import - fully automatized user *inclusion requests*/*file submissions* of new ontologies; **DI**: aut. ont. discovery; **UP**: aut. update of ont.; **UV:** unified ont. versioning labels; **SV**: aut. semantic versioning of ont.; **ID**: stable ont. (version) id (IRI); **PE**: persistent ont. version access for id; **OF**: access to ont. in one unified format; **MA**: system ont. metadata access - *REST API*/*SPARQL*; **TE**: flexible aut. testing of ont. consistency and conformity.

^1^account/login required; ^2^per ontology setting; ^3^imported repos not in sync anymore; ^4^reported, not accessible; ^5^depending on used mvn repository systems; ^6^not working due to missing void file

Archivo is the only system offering a fully automatically processed and invokable user inclusion request for an ontology (LOV requires a thorough review by its community). Apart from LOV, which analyzes referenced ontologies, none of the systems implemented a strategy to discover and include further ontologies or even use multi-layered approaches like Archivo. Besides OBO foundry and OntoMaven relying on a push-only approach, all systems use an automatic fetc.h (update) mechanism to serve the latest version of an ontology. Archivo is the only system providing Semantic Versioning and guaranteeing fully automatic unified versioning, whereas Bioportal and LOV try to extract unified timestamp versioning metadata but also partially rely on correct user input, OBO f. has a publishing principle for unified versioning, which is aut. verified but seems not enforced (review revealed non-uniform versioning labels). With regard to ontology citation or dependency management of ontologies, Archivo and OntoMaven (we were not able to find any hosted ontology though) qualify by providing unified and stable, abstract identifiers (independent of the archiving system and ontology serialization) for ontologies and its version, while taking extra effort to achieve persistent access to the ontology for these identifiers. Besides Bioportal all systems try to reduce the variety of ontologies by supplying every ontology in at least one unified format. Versioning/ontology system metadata access for Archivo is designed to work via RDF and SPARQL, at the time of writing there is only a very basic REST API (and Linked Data interface) available. Both OBO f. and Archivo leverage a continuous, flexible/customizable testing system which is coordinated and performed at a central place to report issues and improve quality, in contrast to Ontohub and OntoMaven focussing on custom tests from/for publishers.

The comparison clearly shows that Archivo addresses a gap and is, to the best of our knowledge, the only system which tries to tackle the (most) user challenges at web-scale and a consumer can rely on that the archived ontology retrieved by a timestamp version resolves to the one that had been served by the ontology authority/domain at that time (no uploader hijacking and curator errors possible).

## Future Work

On a conceptual level, we would like to develop Databus mods^21^ further in order to allow users to augment the archived ontologies with modular contributions (e.g. labels for another language, mappings, another validation report, custom star ratings, etc.). This could strengthen the idea of a platform economy - users contribute what they are in need of for other users. From a technical perspective we plan to implement the Memento protocol for the Databus/Archivo and offer ontology publishers to use Archivo as “plug and play Memento as a service” for their ontologies, to support adoption of Memento and to not take away URI ownership and traffic from the publishers. We also plan to integrate more existing ontology repositories to increase the coverage for other domains. We aim to further enhance existing Databus tools, such that they improve support for special aspects of ontology consumption (e.g. automatic client side conversion of ontology formats and ontology import dependency rewriting with Databus client).

## Footnotes

1 2

3 https://archive.org/web/.

4 https://www.w3.org/DesignIssues/LinkedData.html.

5 https://lod-cloud.net/.

6 https://lov.linkeddata.es/Recommendations_Vocabulary_Design.pdf.

7 https://databus.dbpedia.org/ontologies.

8 13 years in existence, backed up by libraries (TIB) and universities (Mannheim) who are DBpedia Association members.

9 https://www.w3.org/TR/shacl/.

10 http://xmlns.com/foaf/spec/ Supplement: https://github.com/dbpedia/Archivo/tree/master/paper-supplement.

11 The subject of the license statement is the HTML document.

12 cf. Sect. 4.5 of VoID: https://www.w3.org/TR/void/#class-property-partitions.

13 http://archivo.dbpedia.org/add.

14 https://github.com/stardog-union/pellet.

15 http://archivo.dbpedia.org/info?o=.

16 http://librdf.org/raptor/rapper.html.

17 https://linux.die.net/man/1/comm.

18 http://oops.linkeddata.es/catalogue.jsp.

19 OBO, http://obofoundry.org/principles/fp-000-summary.html, link to automated checks.

20 SHACL test https://github.com/dbpedia/Archivo/blob/master/shacl-library/license-I.ttl.

21 http://dev.dbpedia.org/Databus_Mods.

## References

[1] Alobaid, A., Garijo, D., Poveda-Villalón, M., Santana-Pérez, I., FernándezIzquierdo, A., Corcho, Ó.: Automating ontology engineering support activities with OnToology. J. Web Semant. **57** (2019)

[2] Arndt N, Naumann P, Radtke N, Martin M, Marx E (2019). Decentralized collaborative knowledge management using Git. J. Web Semant..

[3] Codescu M, Kuksa E, Kutz O, Mossakowski T, Neuhaus F (2017). Ontohub: a semantic repository engine for heterogeneous ontologies. Appl. Ontol..

[4] d’Aquin M, Noy NF (2012). Where to publish and find ontologies? A survey of ontology libraries. J. Web Semant..

[5] Frey J, Hofer M, Obraczka D, Lehmann J, Hellmann S, Ghidini C (2019). DBpedia flexifusion the best of wikipedia $$>$$ Wikidata $$>$$ your data. The Semantic Web – ISWC 2019.

[6] Halilaj L, Blomqvist E, Ciancarini P, Poggi F, Vitali F (2016). VoCol: an integrated environment to support version-controlled vocabulary development. Knowledge Engineering and Knowledge Management.

[7] van Harmelen F, Klusch M, Rovatsos M, Payne TR (2006). Semantic web research anno 2006: main streams, popular fallacies, current status and future challenges. Cooperative Information Agents X.

[8] Jackson, R.C., Balhoff, J.P., Douglass, E., Harris, N.L., Mungall, C.J., Overton, J.A.: ROBOT: a tool for automating ontology workflows. BMC Bioinform. **20**(1), 407:1–407:10 (2019) 10.1186/s12859-019-3002-3 PMC6664714 31357927

[9] Janowicz, K., Hitzler, P., Adams, B., Kolas, D., Vardeman, C.: Five stars of linked data vocabulary use. Semant. Web **5** (2014)

[10] Jupp, S., Burdett, T., Leroy, C., Parkinson, H.E.: A new ontology lookup service at EMBL-EBI. In: SWAT4LS (2015)

[11] Meinhardt, P., Knuth, M., Sack, H.: TailR: a platform for preserving history on the web of data. In: SEMANTiCS (2015)

[12] Ong, E., Xiang, Z., Zhao, B., Liu, Y.: Ontobee: a linked ontology data server to support ontology term dereferencing, linkage, query and integration. Nucleic Acids Res. **45**(D1) (2016) 10.1093/nar/gkw918 PMC5210626 27733503

[13] Paschke A, Schäfermeier R, Nalepa GJ, Baumeister J (2018). OntoMaven - maven-based ontology development and management of distributed ontology repositories. Synergies Between Knowledge Engineering and Software Engineering.

[14] Peroni S, Shotton D, Vitali F, Teije A (2012). The live OWL documentation environment: a tool for the automatic generation of ontology documentation. Knowledge Engineering and Knowledge Management.

[15] Polleres A, Kamdar MR, Fernández JD, Tudorache T, Musen MA (2020). A more decentralized vision for Linked Data. Semantic Web.

[16] Poveda-Villalón M, Gómez-Pérez A, Suárez-Figueroa MC (2014). OOPS! (OntOlogy Pitfall Scanner!): an on-line tool for ontology evaluation. IJSWIS.

[17] Roussakis, Y., Chrysakis, I., Stefanidis, K., Flouris, G.: D2V: a tool for defining, detecting and visualizing changes on the data web. In: ISWC P&D. CEUR Workshop Proceedings (2015)

[18] Smith B, Ashburner M, Rosse C, Bard J (2007). The OBO foundry: coordinated evolution of ontologies to support biomedical data integration. Nat. Biotechnol..

[19] de Sompel, H.V., Sanderson, R., Nelson, M.L., Balakireva, L., Shankar, H., Ainsworth, S.: An HTTP-based versioning mechanism for linked data. In: LDOW. CEUR Workshop Proceedings (2010)

[20] Vandenbussche P, Atemezing G, Poveda-Villalón M, Vatant B (2017). Linked Open Vocabularies (LOV): a gateway to reusable semantic vocabularies on the Web. Semant. Web.

[21] Vander Sande, M., Verborgh, R., Hochstenbach, P., Van de Sompel, H.: Toward sustainable publishing and querying of distributed Linked Data archives. J. Doc. (2018)

[22] Völkel, M., Groza, T.: SemVersion: an RDF-based ontology versioning system. In: ICWI (2006)

[23] Whetzel PL, Noy NF, Shah NH, Alexander PR (2011). BioPortal: enhanced functionality via new Web services from the NCBO to access and use ontologies in software applications. Nucleic Acids Res..

[24] Wilkinson MD, Dumontier M, Aalbersberg IJ, Appleton G (2016). The FAIR Guiding Principles for scientific data management and stewardship. Sci. Data.

